# Aggregate Effect on the Concrete Cone Capacity of an Undercut Anchor under Quasi-Static Tensile Load

**DOI:** 10.3390/ma11050711

**Published:** 2018-05-01

**Authors:** Marco Marcon, Krešimir Ninčević, Ioannis Boumakis, Lisa-Marie Czernuschka, Roman Wan-Wendner

**Affiliations:** Christian Doppler Laboratory LiCRoFast, Department of Civil Engineering and Natural Hazards, University of Natural Resources and Life Sciences (BOKU), 1190 Vienna, Austria; mmarco88@gmail.com (M.M.); nincevick@gmail.com (K.N.); ioannis.boumakis@boku.ac.at (I.B.); lisa.czernuschka@boku.ac.at (L.-M.C.)

**Keywords:** concrete, fasteners, aggregates, undercut anchor, re-sampling, photogrammetry

## Abstract

In the last decades, fastening systems have become an essential part of the construction industry. Post-installed mechanical anchors are frequently used in concrete members to connect them with other load bearing structural members, or to attach appliances. Their performance is limited by the concrete related failure modes which are highly influenced by the concrete mix design. This paper aims at investigating the effect that different aggregates used in the concrete mix have on the capacity of an undercut anchor under tensile quasi-static loading. Three concrete batches were cast utilising three different aggregate types. For two concrete ages (28 and 70 days), anchor tensile capacity and concrete properties were obtained. Concrete compressive strength, fracture energy and elastic modulus are used to normalize and compare the undercut anchor concrete tensile capacity employing some of the most widely used prediction models. For a more insightful comparison, a statistical method that yields also scatter information is introduced. Finally, the height and shape of the concrete cones are compared by highly precise and objective photogrammetric means.

## 1. Introduction

Compared to cast-in connection details, post-installed anchors provide the opportunity to design modular structures, accelerate the construction process and renovate or strengthen old buildings. In consequence, the usage of post-installed fastening systems has been increasing steadily during the last decades. Depending on the specific anchor system and load situation, several different failure mechanisms ranging from yielding of steel, failure of the adhesive interface or progressive fracture of the concrete substrate, leading to the so called “concrete cone break-out”, can occur. For the latter, several equations (see e.g., [1,2,3]) have been proposed. These equations attempt to cover, in a semi-empirical fashion, a failure mode that is most clearly observed for cast-in headed stud anchors. The failure is the result of a propagating crack (starting at the anchor head) and ultimately loss of equilibrium.

Cast-in, as well as post-installed anchors, have been studied extensively with experimental and numerical means to identify the complex failure mechanism and its influencing factors, among them the concrete properties, the anchor geometry, and test configuration. Regarding the concrete properties, many studies have been performed showing differences in terms of anchor capacity for normal and high strength concrete [4]. Also, experiments on anchors installed in fiber reinforced concrete were carried out [5] in order to quantify the performance improvement of fasteners installed in a more ductile base material. Another way to increase the performance of cast-in anchors has been presented by Turker at al. [6] by introducing studs surrounding the anchor.

Since fasteners are known to attract or induce cracks, the behaviour of pre- and post-installed anchors have been studied extensively both in plain and cracked concrete (see e.g., [7]). Concrete age also influences the concrete mechanical properties. For this reason, researcher analysed the evolution of the concrete cone failure load in time, especially at early age [8] since there are application scenarios in which the anchor needs to be loaded as early as 18 h after casting.

Another influencing factor is the anchor geometry which has been studied in order to relate to the anchor’s features, not only the peak load, but also the global behaviour. For instance, Nilforoush et al. analysed the influence of the headed stud head size and the concrete slab thickness. They related their experimental results to the predictions obtained by equations [9,10]. In these contributions the scatter of the experimental results was significantly reduced by accounting for the ratio of bearing stress (local stress acting on the anchor head) at peak load to the concrete compressive strength. Following a similar goal, i.e., improving the understanding of the experimental scatter, this paper aims at verifying or disproving the hypothesis that the type of (coarse) aggregate used affects the concrete cone capacity beyond what can be accounted for using the current prediction equations.

With the rise of computational power increasingly more important insights are gained by simulations. Artificial Neural Networks (ANN) have been successfully used to predict the capacity of anchors loaded in tension [11] or shear [12] based on a set of influencing parameters that have been chosen during the training phase of the network. In general, numerical analysis is a wide spread method to simulate the behavior of anchors installed in concrete in order to support, or sometimes extend, experimental results. Simulations were performed to improve the understanding of anchors installed in normal concrete [13,14], lightweight concrete [15], or in the presence of reinforcements [9,10]. Additionally, simulations were run to investigate different loading directions [16,17,18], loading rates [19], and temperatures [20,21].

Finally, also the influence of the concrete properties on the anchor capacity has been numerically analysed [13]. Compressive and tensile strength, fracture energy and head diameter were varied in this study. Results showed, for this specific anchor geometry, that the main parameter which influences the peak load is the fracture energy, while the other parameters have less influence on the peak but relevant influence on the global behaviour. In the case of mechanical anchors, speculations on the reason for the large experimental scatter have been published by many researchers. Among those, the “aggregate effect” is one of the most widely cited reason which, however, has not been systematically studied up to now, especially for undercut anchors.

In order to maximise consistency between laboratories, and cover all relevant cases, specific guidelines were created [22,23,24,25,26]. Even though the ultimate product qualification is product specific, and should be independent of the certified lab, anchors are always tested together with the substrate material which in most cases is concrete. Only in few cases, researchers were able to derive functional replacement tests (see [27]) that can be fully standardised and are independent of the base material. Most of the qualification tests are performed on anchors installed in a concrete member which was produced by a local supplier. It is well known that concrete from different places can exhibit different properties, stemming from differences in the constituents. Not only can the concrete mix vary, but also the properties of different cement types, or aggregates, can exhibit substantially different properties from area to area.

The concrete cone capacity is influenced by many parameters already under quasi-static conditions, not considering further loading and deterioration scenarios. For this reason, three concrete batches were cast utilising different aggregates but maintaining the overall mix design in an attempt to isolate a potential “aggregate effect” on the concrete cone failure of an undercut anchor product found on the market, as reported earlier [28]. All the other influencing parameters such as curing protocol, testing age, loading rate, mechanical boundary conditions during test, slab geometry, and person installing the anchor were kept constant. Also the drill bit was replaced for each batch and age. For each of the three batches, slabs were produced for the anchor pull-out tests, and small scale specimens (cubes, cylinders and beams) for the material characterisation. In this study, all concretes have been fully characterised in terms of elastic modulus, tensile and compressive strength, and fracture properties. After installing the undercut anchors in the slabs they were tested under quasi-static tensile loading only. All tests on the material and system scale were performed 28 and 70 days after casting. All failure surfaces were documented by photogrammetry.

This comprehensive dataset allows the direct investigation of correlations between material properties and system response. Furthermore, this data can also serve for the calibration of material parameters in numerical investigations which will be used to gain more insights into the problem, and/or extend numerically the experimental dataset (as done in [29]).

After a short review of the concrete cone capacity design (CCD) method, the test results of the three different batches are systematically analysed and compared in terms of (a) depth and shape of the crater left in the slab after ripping out the concrete cone, and (b) the undercut anchor capacity in tension normalized by the concrete properties. The link between available concrete material properties and system response is studied utilising the currently established analytical prediction models.

## 2. Review of State of the Art

This manuscript deals with an undercut anchor product which is placed in unreinforced concrete and tested under pure tensile load in a configuration where the anchor response at peak load is not affected by the supports. In the fastening community, this set-up is referred to as “unconfined configuration”. In general, pull-out tests can be considered to be unconfined (see [30]) when the concrete slab is supported with a distance $s_{width}>3\;h_{ef}$, where $h_{ef}$ is the embedment depth of the undercut anchor and $s_{width}$ is the support distance. Under these conditions a concrete cone failure mechanism starting at the anchor head can be expected for the investigated anchor type.

Other failure mechanisms in tension, like steel failure and pull-through failure [2], are relevant for fastenings but will not be investigated in this paper, neither will be concrete splitting, edge failure [31], and pry-out failure [32]. The tests have been designed accordingly to trigger only concrete cone failure.

During the past years, solid design methods have been developed for fastening systems. Fuchs et al. introduced a design procedure, the concrete capacity design approach (CCD method, see [2]), which is now the state of the art in fastening systems design. The CCD method allows designing single anchors as well as anchors groups, and is based on the behaviour of a single anchor, subjected to shear, tensile or combined load. The CCD method originates from an equation proposed by ACI Committee 349 to estimate the load capacity of a mechanical anchor [1]. Equation (1), at that time, has been recommended for the estimation of the concrete breakout failure. The equation works for a single mechanical anchor under tensile load, placed in un-cracked and unreinforced concrete, not influenced by edges and in an unconfined situation.

(1) $$P_{d}=4\cdot \phi \cdot \sqrt{f_{c}}\cdot A_{p}$$

Equation (1) is based on plasticity theory and states that the pull-out concrete strength $P_{d}$ is based on an uniform tensile stress equal to $4\phi \sqrt{f_{c}}$ (where $f_{c}$ is the concrete compressive strength and ϕ is a reduction factor) acting on an effective area $A_{p}$. The projected area of the stress cone can be calculated using an inclination angle of 45${}^{\circ }$ from the anchor head. The load is introduced locally at the lower end of the anchor and causes high compressive stresses. Global equilibrium is achieved through a tensile stress field concentrated at the anchor head. Failure occurs after the tensile strength is exceeded and the developing crack propagated a certain distance at which point the equilibrium cannot be satisfied any more. Consequently, concrete cone failure is a fracture mechanics problem, best described through tensile strength and fracture energy and, thus, it is also subject to size effect. If the fracture process zone is small compared to the structure size then, with good approximation, a stress singularity can be assumed and linear elastic fracture mechanics (LEFM) applies (see [33]). Compared to cohesive fracture mechanics with a finite process zone, LEFM is associated with the strongest possible size effect with exponent −0.5. The fracture mechanics based size effect for fastenings has been experimentally and theoretically verified in several investigations (see [3,34]) and is widely accepted, also due to the fact that, in this case, LEFM is a conservative and safe choice. A more detailed explanation of the application of Bažant’s size effect theory on fastenings is given in [35]. Introducing the LEFM size effect in Equation (1) yields a predictive equation for the anchor capacity in tension with three calibration factors.

(2) $$N_{no}=k_{1}\cdot \sqrt{f_{c}}\cdot k_{2}\cdot h_{ef}^{2}\cdot k_{3}\cdot h_{ef}^{-0.5}$$

For practical purposes this equation can be simplified to:

(3) $$N_{no}=k\cdot \sqrt{f_{c}}\cdot h_{ef}^{1.5}$$

The CCD method formulation according to Equation (3) estimates the pull-out load $N_{no}$ (in N) based on the compressive strength of the concrete obtained from a 200 × 200 mm cube, $f_{c}$ (in MPa), the embedment depth of the anchor, $h_{ef}$ (in mm), and a parameter *k* which is derived as product of three factors $k_{1}$, $k_{2}$, and $k_{3}$. Eligehausen at al. proposed an alternative formulation which uses the fracture energy and the elastic modulus of concrete in order to obtain a more accurate prediction equation, e.g., for research [3], by applying an energetic model. The crack starts when the crack formation energy G(w) is reached, which depends on the tensile strength $f_{t}$ and the fracture energy $G_{F}$ [36]. From the variation of the total free energy with the crack length and angle, a relation can be derived between the relative crack length and the load. The resulting relation is Equation (4) which includes a function $n(cl/cl_{max})$ that relates the load to the ratio between the relative progress of the crack cl and the total crack length $cl_{max}$ that the crack would have when it reached the surface with a slope of 37.5${}^{\circ }$.

(4) $$N_{no}=n\cdot \sqrt{E\cdot G_{F}}\cdot h_{ef}^{1.5}$$

The function *n* reaches a maximum of 2.1 at a relative crack length of around 40%. Consequently, the concrete conce capacity of the anchor under tensile load can be obtained according to Equation (4) with n=2.1.

This equation though, is not widely used because it is based on fracture energy which is difficult to obtain experimentally and is normally affected by high scatter.

From a regression point of view, the best model is the one that represents a given phenomena with the least number of independent variables. Independent variables carry more informations than correlated ones as shown for instance in [37] for the principle component analysis. For concrete, correlation matrices have been proposed in the literature to describe the mutual dependence of concrete material properties (see [38,39,40,41]). Considering fracture energy and elastic modulus, Equation (4) contains the two most uncorrelated concrete parameters, making it, potentially, the most eligible equation.

## 3. Aggregate Effect on Concrete Properties

The local availability influences the choice of the aggregate type used for the concrete production. As a result, used aggregates present differences in mineralogical composition, dimension and shape. In the literature, an extensive amount of studies concerning the aggregate effect on concrete can be found. For instance, Delmar et al. [42] investigated the influence of coarse aggregates on compressive strength and elastic modulus of high performance concrete. They concluded that smaller coarse aggregates yield higher compressive strength, especially for lower water-cement ratios. Furthermore, the influence of mineralogical type of aggregate was studied by Ke-Ru and co-workers (see [43]) by testing concretes with different coarse aggregates and water cement ratios. They found that the effect of aggregate is more pronounced for high strength concrete especially on compressive strength and fracture energy and that the influence on tensile strength is minor. As mentioned before, also the shape of coarse aggregates plays a major role in the behaviour of concrete, especially for fracture mechanical purposes. For that reason, Guinea et al. [34] studied the matrix-interface influence on macroscopic fracture parameters testing concrete mixes with different coarse aggregate shapes. Results showed that concretes with broken aggregates, in general, show higher fracture energies due to mechanical interlock.

Concrete is a heterogeneous composite material. In spite of several decades of research, prediction models that link the mechanical properties of concrete constituents to those of the composite are still largely missing. Consequently, in this contribution the concrete cone capacity of the investigated undercut anchor product is directly related to the mechanical properties of the concrete (which are influenced by the used aggregates).

### Aggregate Effect Based Anchors Working Principles

As just discussed, aggregate type, shape and size influence the concrete behaviour. Thus, an influence on the system behaviour can also be expected. The influence that the aggregates can have on the undercut anchor tensile capacity can be divided into two categories: (a) the direct influence on the concrete properties and consequently on the system behaviour; and (b) an indirect influence of the aggregates on the system behaviour that is not related to changes in concrete properties.

In the second case, different aggregates would assert further influence on the system capacity and this influence would depend on the working principle of the considered anchor type. Typical working principles of anchors are interlock, friction and adhesion. In the specific case of the investigated undercut anchor product, the working principle is primarily interlock which is obtained by creating an undercut with a special drill bit. The drilling procedure and resulting undercut can be influenced by the aggregate type. Due to the heterogeneity of concrete, a drilled hole or undercut will rarely be perfectly axisymmetric, leading to a certain degree of uncertainty on the undercut geometry and on the resulting embedment depth. After the borehole is drilled, the anchor is installed and pre-tensioned. In this process the anchor is forced to adapt its shape to the previously generated undercut, establishing the actual mechanical interlock and embedment depth. Said uncertainty is addressed in Section 6 by measuring the real concrete cone depth and its variability via photogrammetry.

## 4. Experimental Campaign

The experimental campaign includes tests for the characterisation of the concrete material properties and unconfined pull-out tests on the investigated undercut anchor product in order to determine the anchor capacity in tension. Additionally, the used coarse aggregates were mechanically characterised by means of micro-indentation. For each of the three batches, three repetitions for each standard specimen (cube, cylinder, prism) were tested, and five pull-out tests were performed. Slabs of 1.5 × 3.0 × 0.3 m were used for the study in order to (a) guarantee enough space for unconfined tests, and (b) avoid influences from the edges. All the tests were performed for both investigated concrete ages, namely 28 and 70 days.

**Figure 1:**
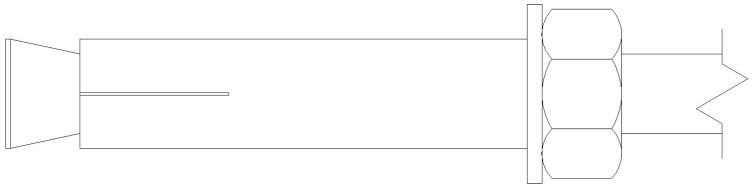
Qualitative representation of the undercut anchor used in the experimental campaign.

A 100 mm long post-installed undercut anchor is investigated in the present study. In a first step, a cylindrical hole opening up in a conical undercut is created with a special drill bit. After inserting the anchor consisting of a metal sleeve and a threaded bar ending in a cone, a given installation torque is applied. In this process the anchor adapts to the conical void by which mechanical interlock is achieved. A qualitative sketch of the undercut anchor is shown in Figure 1. The anchor complies with the requirements given in [24].

The concrete craters left in the slabs after performing the pull-out tests have been digitalized by photogrammetric means in order to be able to comprehensively investigate the aggregate influence including the geometry of the failure surface.

### 4.1. Mix Design

In order to investigate the potential aggregate effect on the undercut anchor capacity in tension, three concrete mixes were tested. These three concrete mixes, identified as D1, D2 and D3, were class C25/30 and had the same cement type CEM II 42.5 N. The concrete was delivered by a ready-mix concrete company which optimized also the mix design based on the requirement to alternate the (coarse) aggregate (type, size, mechanical properties) in the widest range possible while maintaining the rest of the mix design. Table 1 shows the details of the mix design used for the three concretes. Water and cement content are very consistent among the three batches resulting also in a constant w/c value. To all the batches, 1.40 kg/m${}^{3}$ of plasticizer and 0.27 kg/m${}^{3}$ retarder have been added to ensure workability over the full casting duration.

### 4.2. Curing Conditions

According to the approval guidelines for mechanical anchors [30], the concrete specimens used to obtain the mechanical properties, as well as the concrete slabs for the anchor system tests have to be cured together for seven days in a closed environment. After that, they can be stored also in an open environment if properly protected from freeze and thaw. Other standards, especially related to testing material properties, require curing concrete specimens in a water bath or in a moist curing room with a humidity of at least 95% (see [44]).

It is commonly known that concrete already approaches it’s full strength at 28 days. In case of the cement used in this experimental campaign, the 28 days compressive strength is around 82% of the asymptotic value at full hydration, if the fib model-code equation ([45]) is used. After that though, the hydration continues for months at a continuously decelerating rate [46]. Thus, a further change in mechanical properties and concrete tensile anchor capacity can be expected.

Two curing protocols have been considered for the small scale specimens (specimens used for the concrete characterization): (a) stored with the slab according to the fastenings related guidelines denoted as D (as dry); and (b) stored in lime saturated water until the testing day denoted as M (as moist).

Compression tests were performed on cubes for the determination of compressive strength ($f_{c}$) and on cylinders for the elastic modulus (*E*). The moist cure condition was used for cylinders and part of the cubes, and the dry cure condition was used just for the remaining cubes. Fracture tests were performed on moist cured notched beams in a three point bending configuration in order to obtain the total fracture energy ($G_{F}$) and the initial fracture energy ($G_{f}$). Indirect tensile strength ($f_{t,sp}$) was obtained from Brazilian splitting tests. The splitting specimens were stored according to the moist protocol. The slabs were stored according to the fastenings related guidelines which corresponds to dry curing according to the above mentioned definition.

## 5. Experimental Results

### 5.1. Aggregate Properties

The three different aggregate types used in this campaign are Quartz, Limestone and Basalt. Elastic moduli and Vickers hardness have been obtained for the three aggregate types. The Quartz aggregates have an elastic modulus of 11.4 GPa and a Vickers hardness of 0.417 GPa. The Limestone aggregates have an elastic modulus of 37.5 GPa and a Vickers hardness of 1.26 GPa. The Basalt aggregates have an elastic modulus of 45.6 GPa and a Vickers hardness of 2.08 GPa.

### 5.2. Concrete Properties

Concrete properties were experimentally determined at 28 and 70 days. The elastic modulus was extracted from the loading branch of the cylinder compression tests. The deformation has been measured using three LVDTs (linear variable differential transformer) with a base length of 100 mm, placed parallel to the specimen axis, in a 120${}^{\circ }$ configuration, and centered along the specimen height. The results are available in Table 2. The aspect ratio of the cylinders, defined as the ratio between length (*L*) and diameter (*d*), is L/d=2 (where L=300 mm and d=150 mm). The modulus of elasticity is very consistent within the batches at 70 days both in terms of mean value and in terms of coefficient of variation (COV). At 28 days the D2 concrete exhibits a lower elastic modulus in comparison with the D1 and D3 concretes.

Compression tests were performed on cubical specimens with an edge length of 150 mm for the two different curing conditions, as shown in Table 2. From the table, it can be seen that dry cubes have lower strengths compared to the moist cured ones. Since dry cured specimens are not exposed to a moisture saturated environment, they can suffer from incomplete curing due to the reduced water availability, and also from drying shrinkage damage.

Fracture energy is defined as the energy required to create a unit area of crack surface and it is an important property of concrete for investigations concerning fracture and damage propagation. The total fracture energy $G_{F}$ (shown in Table 2) is determined as the ratio between work of fracture and specimen ligament area. A stable test is needed to obtain a result where the load approaches zero load level in the softening branch. When this is not possible, the curve can be extrapolated with e.g., an exponential function. Alternatively, other techniques based on inverse analysis can be employed such as e.g., the sequentially linear analysis [47]. The initial fracture energy $G_{f}$ (shown in Table 2) is approximated by integrating the area under the linear fit to the beginning of the softening load displacement curve as suggested by [48]. The resulting area is again divided by the ligament area yielding a property which is less influenced by the experimental set-up of the fracture test and that can be a more reliable concrete parameter in comparison with the total fracture energy. These properties were obtained from three-points bending tests on notched beams having dimensions 100 × 100 × 400 mm, a notch depth of 30 mm, and a span of 300 mm between the lower supports. The COV of the fracture properties is higher than all the other properties, as expected. Fracture tests are available just for the concrete age of 70 days because of technical problems with the load frame at 28 days.

Tensile strength was also obtained experimentally. Brazilian splitting tests were performed on cylindrical slices having a diameter of 150 mm and length of 75 mm. Between the loading plates and the specimen, wooden strips were placed to distribute evenly the load. Such results are available in Table 2.

### 5.3. Anchor Capacity Experimental Results

The results of the pull-out tests of the undercut anchors used are also summarised in Table 2 for both ages. The mean values of the anchor tensile capacity tend to increase for the older concretes. The COV is lower than the maximum value allowed by the relevant guidelines. The difference in the anchor capacities in tension should come from the direct effect of the aggregates on the material properties of the concrete (compressive strength, elastic modulus and fracture energy). In order to evaluate the presence of an indirect effect of the aggregates on the tensile capacity of the undercut anchor, the experimental maximum pull-out load has to be normalised by the material properties utilising one of the established predicting equations.

## 6. Photogrammetric Measurement of Crater Depth

The 3D geometrical representation of the crater left from the concrete cone failure is acquired through a photogrammetric tool yielding a 3D point cloud. In order to obtain the crater shape, several pictures have to be taken from different distances and positions. A series of targets are placed around the area to determine the scale and the coordinate system, and to facilitate the recognition of common points among pictures.

Once the point cloud is acquired, the cone is divided into slices (here 36 slices of 10${}^{\circ }$) and for each of them the crater depth is determined. The individual slice estimates are then averaged to obtain the estimated crater depth.

**Figure 2:**
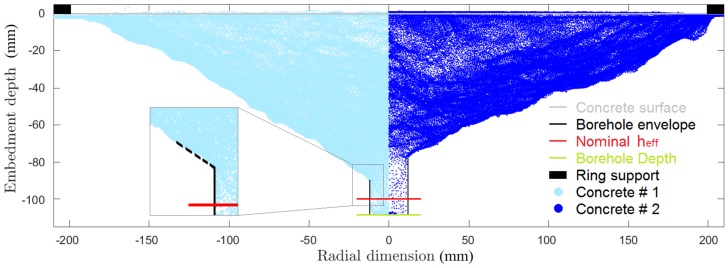
Comparison of point clouds acquired for two craters and test set-up representation.

For the crater depth estimate, each individual slice is plotted in a diagram of embedment depth (*y*-axis, vertical dimension) versus radius (*x*-axis, radial dimension). Points having a radial coordinate value within 15 mm and 30 mm serve as data basis for linear regression (as shown in Figure 2 by the dashed black line). The measured crater depth is obtained as intercept of the linear extrapolation with the nominal anchor diameter. Figure 2 exemplarily shows the difference between two concrete craters and the information related to the set-up (supports, borehole depth and nominal embedment depth).

**Figure 3:**
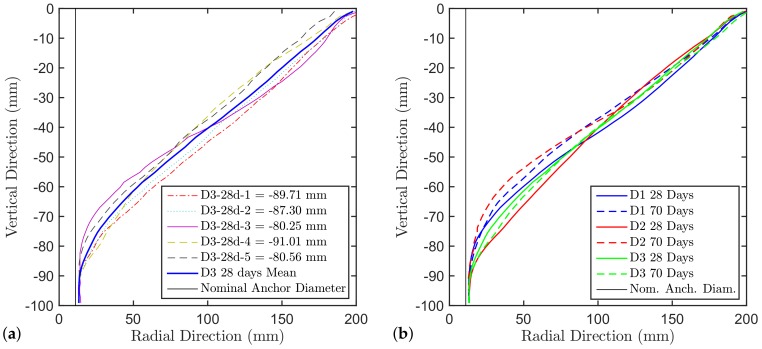
(**a**) Mean profiles of the craters of D3 at 28 days and their mean, and (**b**) comparison of the mean crater profiles of the different batches and different ages.

Figure 3a shows the individual mean crater profiles of the D3 concrete at 28 days as an example, while Figure 3b shows the comparison of the mean crater profiles of the different batches and different ages. From the latter it can be seen that generally the crater is shallower at 70 days compared to 28 days even though the concrete tensile capacity is higher. All the crater profiles converge on one end to the support ring and on the other end to the undercut (excavated by the drill bit) which was destroyed during the test. Note, although the cones converge to the support ring, no effect on the pull-out capacity can be expected since the support distance in this study was chosen to be $4\;h_{ef}$. This is larger then the code requirement of $3\;h_{ef}$ for unconfined tests supported by many experimental studies. The reason is that the peak load in a pull-out test is reached well before the crack approaches the surface (when the relative crack length approaches about 40% of the distance to the surface). Above $3\;h_{ef}$ there is no noticeable influence of the boundary conditions on the stress field governing the behavior up to the peak load.

Hence, only the post-peak branch of the load displacement curve and the crack path in the softening regime will be influenced by the ring.

**Figure 4:**
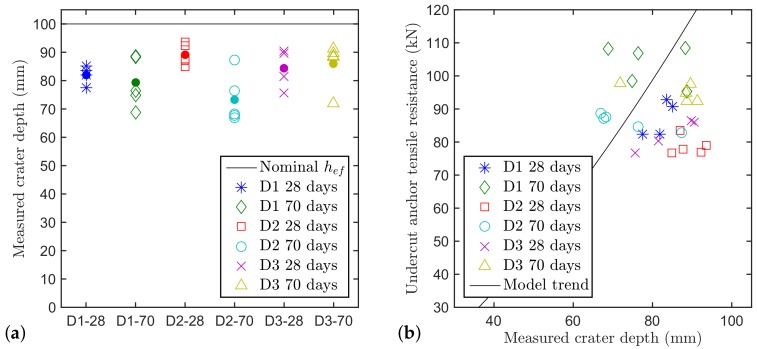
(**a**) Measured concrete crater depth (dots represent the average of the relative group of results); and (**b**) correlation between anchor tensile capacity of a single undercut anchor and its crater depth.

The crater depth and shape are different among the batches due to the different effect that aggregates have on the concrete mechanical properties. The borehole, and especially the undercut, as already mentioned, can have different shape depending on the resistance that the concrete poses to the drilling procedure and the expansion of the anchor during the pre-stressing procedure. Figure 4a shows the measured crater depths compared to the nominal one. From the figure, it can be seen that the measured crater depth is around 20% smaller than the nominal embedment depth and that different aggregate types are associated with different crater depths. The measurements, though, show a quite large scatter due to the concrete heterogeneity.

According to the design equation, the capacity of the undercut anchor in tension should grow with $h_{ef}^{1.5}$ (shown in Figure 4b as trend line). The hypothesis of a perfect cone naturally is a convenient empirical approximation. This concrete cone is dependent only on the embedment depth and has a fixed angle of around 35${}^{\circ }$.

Figure 4b shows the correlation between the failure loads and the measured crater depth. There is no apparent correlation between pull-out load and measured crater depth (at least for the available crater depths). This indicates that the tensile resistance is indeed not only determined by the localised crack but also by product specific local effects around the anchor head. For this reason, in this paper the nominal value of the embedment depth will be used for all the following analyses as opposed to the real crater depth.

## 7. Normalisation of the Undercut Capacity in Tension

In order to investigate the presence of an additional aggregate effect on the concrete tensile capacity of this undercut anchor product, the experimental pull-out results are compared to the predictions by the established models accounting for the material properties at the age of the anchor test. For that purpose the experimental results ($N_{exp}$) are normalised by dividing with the predicted pull-out load ($N_{mod}$), calculated either by Equations (3) or (4). The normalised pull-out load can be defined as $\alpha_{fc}$ or $\alpha_{Gf}$ depending on which concrete properties are used in the prediction model.

(5) $$\alpha_{fc}=\frac{N_{exp}}{N_{mod}}=\frac{N_{exp}}{k\cdot h_{ef}^{1.5}\cdot \sqrt{f_{c}}}$$

(6) $$\alpha_{Gf}=\frac{N_{exp}}{N_{mod}}=\frac{N_{exp}}{n\cdot h_{ef}^{1.5}\cdot \sqrt{E\cdot G_{F}}}$$

The normalisation should isolate the aggregate effect on the pull-out loads from the concrete properties (the anchor features in this case are constant throughout the experimental campaign). In case of a perfect prediction model, the normalised pull-out load would approach 1, while a number bigger than 1 indicates a conservative model. The normalised experimental results themselves do not allow drawing a conclusion regarding the existence of an aggregate effect. Such an interpretation can only be based on the comparison between different batches.

In this investigation, the relative difference RD between the normalised values is introduced. The relative difference is calculated according to Equation (7) with $\alpha_{Di}$ (i=1,2,3) equal to the normalised pull-out load for the three batches and $\bar{\alpha }=$ the respective mean (α stands for either $\alpha_{fc}$ or $\alpha_{Gf}$). The smaller the relative difference RD of the normalized pull-out loads is, the smaller is the additional effect of aggregates on the capacity of the undercut anchor in tension, i.e., the better the design equation can account for the effect that different aggregates have on the anchor capacity. If a big difference is observed, a correction factor to account for the aggregate effect may be derived based on the α parameters used for the comparison. As previously mentioned, the nominal embedment depth is used for the evaluation of all prediction models.

(7) $$RD=\frac{\sqrt{{\left(\alpha_{D1}-\bar{\alpha }\right)}^{2}+{\left(\alpha_{D2}-\bar{\alpha }\right)}^{2}+{\left(\alpha_{D3}-\bar{\alpha }\right)}^{2}}}{\bar{\alpha }}$$

For the mean value analysis just Equation (3) is considered for 28 and 70 days, respectively, since, as previously mentioned, there are no available fracture energy data at 28 days.

### 7.1. Comparison of Mean Values

**Figure 5:**
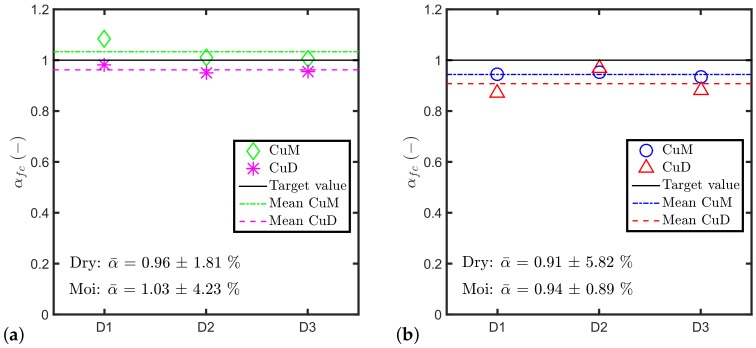
Comparison of $\alpha_{fc}$ parameter among different batches using $f_{c}$D and $f_{c}$M at (**a**) 28 days and (**b**) 70 days.

Figure 5 shows the comparison of the normalised experimental pull-out load $\alpha_{fc}$ parameters for the different batches, using the mean values of the anchor tensile capacity and the mean values of compressive strength of either dry cured cubes ($f_{c}$D) or moist cured cubes ($f_{c}$M). The results presented in the figure suggest that, if there is an additional effect of the aggregates on the undercut anchor tensile capacity, it is very small and may be negligible in practice. For both ages, the relative difference RD among the three batches is smaller than 6%. The result is consistent with the one found in [49] during a study on the dynamic behaviour of mechanical anchors. Also this study concludes that Equation (3) can account for the aggregate type, at least from a practical point of view.

## 8. Statistical Analysis

The fact that in this and most experimental campaigns the number of tests are limited (here to 3 or 5 specimens for each configuration), constrains the certainty of the conclusions if only the mean values are used. Additional insights can be gained by using the information about the scatter in the experiments. Furthermore, it has to be noted that if a function f(x) is non-linear as Equations (3) and (4), $\bar{f(x)}\neq f\left(\bar{x}\right)$. Consequently, the function evaluation based on the mean values of the inputs is only an approximation that is reasonably accurate for most mean value analyses. However, for the evaluation of failure probabilities that are determined by the tails of the distribution this simplification may be insufficient. One way to propagate the uncertainty of the inputs into the prediction is the combination method. Since none of the pull-out tensile capacity values can be related to specific concrete specimen properties, simply all possible combinations are evaluated yielding an estimate of the distribution of normalised pull-out loads $\alpha_{fc}$ and $\alpha_{Gf}$. An example is shown to clarify the combination method. Having i=3 compressive strength results and j=5 pull-out load results, the combination method will provide m=i·j=15 results of $\alpha_{\left(\cdot \right)}$ as shown in Equation (8).

(8) $$\alpha_{\left(\cdot \right)}=\frac{N_{exp}}{N_{mod}}=\frac{N_{exp,j}}{k\cdot h_{ef}^{1.5}\cdot \sqrt{f_{c,i}}}\;\forall \;i=1,2,3\;\forall \;j=1,2,3,4,5$$

With the same procedure, also the combination of the results based on Equation (4) can be obtained. The next paragraph will verify that the combination method provides results which are representative of the original sample.

### 8.1. Increased Sample Size Verification

A verification has been performed in order to confirm that the artificially generated set is representative of the original data set. The dataset is compared with the results of a resampling method which is known to converge to the real solution [50]. The bootstrap method is used to estimate the sampling distribution drawing randomly with replacement from the original data with the purpose of deriving robust estimates of the mean value, standard errors and confidence intervals of a population. The result of this verification is that the first statistical moments of the resampled set converge to those obtained by the combination method. The reason is that if the resampling size tends to infinity, the obtained number of each combination tends to be uniform. In this analysis, it is important to use the population version of the standard deviation (dividing by *n*) and not the sample version (dividing by n−1) because during resampling, the actual observation is considered as the entire population. The advantages of the resampling method are that it is possible to consider correlation between variables and to assign different uncertainties to each observation. Nevertheless, the mean value and standard deviation of the resampled set converge to those obtained by the combination method. The latter being easier and faster was chosen for this study since the drawbacks are not relevant for the investigated problem.

### 8.2. Aggregate Effect Based on Combinations

**Figure 6:**
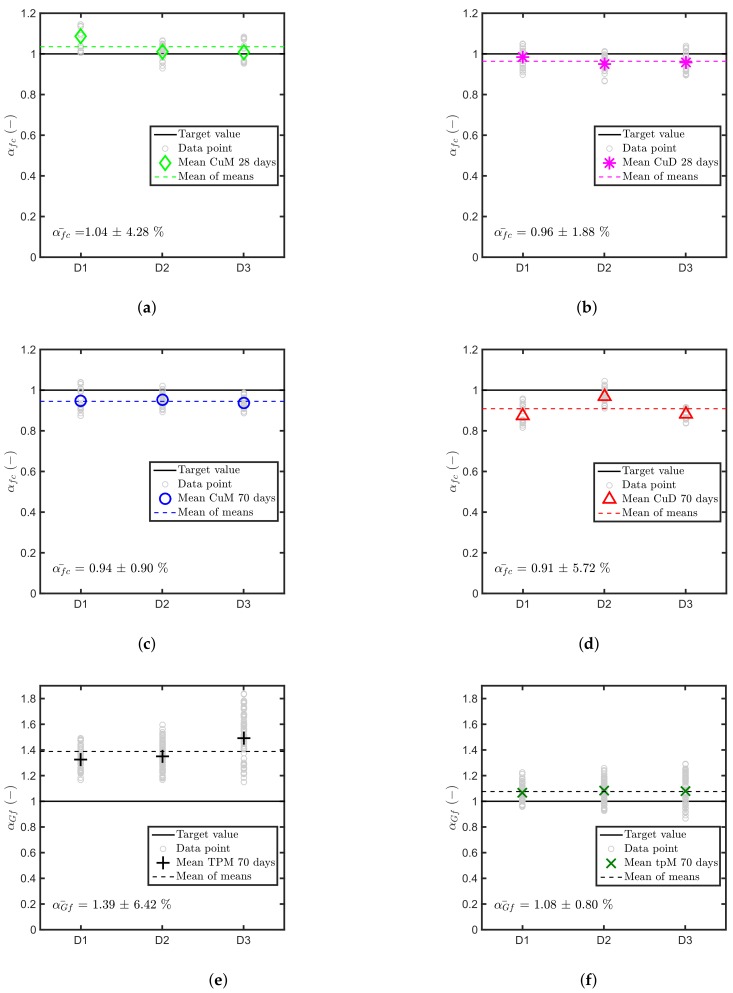
Combinations results of α using Equation (5) with (**a**) $f_{c}$M at 28 days; (**b**) $f_{c}$D at 28 days; (**c**) $f_{c}$M at 70 days; (**d**) $f_{c}$D at 70 days, and using Equation (6) with (**e**) $G_{F}$ and (**f**) $G_{f}$ both at 70 days.

The proposed combinations method introduces the chance to obtain a distribution of normalised pull-out loads (α) rather than a single number. Figure 6a,b show the results of Equation (5) using as concrete properties the compressive strength both for the dry and the moist cases at 28 days. Figure 6c,d show the same results at 70 days. The results of the combination method enforce the mean value analysis results and provide an estimation of the scatter propagated from the test results. It is interesting to note that the scatter within a batch is typically larger than the relative difference between the batches, thus confirming that the “aggregate effect” is negligible, at least from a practical point of view.

Figure 6e,f show the results of the normalisation according to Equation (6) using the combination method for both $G_{F}$ and $G_{f}$. The total fracture energy based prediction overestimates the pull-out load by about 30%. The prediction based on the initial fracture energy, on the other hand, is much closer to the target value. In this figure, by looking at the relative difference RD of the results, the initial fracture energy seems to be able to compensate for the aggregate effect better than $G_{F}$. From the perspective of cohesive fracture mechanics this is in line with the expectations. At the peak load most of the crack, which is propagating starting at the anchor head, has not reached a fully softened state and, thus, the problem is better described by the initial fracture energy.

## 9. Conclusions

In the presented investigation, the potential effect of aggregate type and shape on the concrete capacity of an undercut anchor under tensile loading was investigated experimentally. The experimental results including anchor pull-out tests for three concretes performed at two ages each were presented and compared to the results of a comprehensive material characterisation performed at the same ages.

A photogrammetric tool has been successfully used to gain insights into the failure mechanisms of the studied undercut anchor. Reliable information of the crater depth was obtained for all the batches and ages. With this information it has been possible to show the lack of correlation between the measured crater depth and the anchor capacity in tension of the studied undercut anchor product, highlighting the presence of a load carrying mechanism other than the concrete bearing mechanism. This is true for the studied anchor and for the evaluated embedment depth. To extend this statement, tests on additional embedment depths covering a larger range are required.

The ultimately localised concrete cones for the three investigated concretes differ in shape and depth due to differences in the aggregates and their effect on the concrete properties. However, if the nominal embedment depth, and the undercut anchor related *k* factor (reported in the respective ETA document) are used in the standard prediction Equation (3) based on an idealised concrete cone, the tensile capacity predictions for this undercut anchor are very close to the experimental results and consistent throughout the three investigated concretes. This leads to the conclusion that the aggregates potentially influence the relative contribution of the load carrying mechanisms without affecting the overall performance of the anchor system.

The combination method provides a distribution of the prediction quality parameter α as opposed to a single value. From the obtained distributions, it can be seen that the differences in the normalised results between the batches are generally smaller than the scatter of the measurements in each batch, enforcing the assumption that Equation (3), and also Equation (4), can account for the aggregate effect even though the square root of the compressive strength is solely an empirical predictor for those material properties that determine fracture and, thus, the concrete capacity in tension of the investigated undercut anchor.

The experimental results clearly show that by changing the coarse aggregates both the concrete properties as well as the undercut anchor tensile capacity change. However, based on this experimental campaign, it can be stated that there is no practically relevant aggregate effect on the concrete capacity in tension (for the investigated undercut anchor) beyond what the established model equations can predict. Thus, the current assessment and design framework, which accounts for a variation of the concrete cone capacity as a function of concrete compressive strength only, serves as a safe design method.

## Figures and Tables

**Table 1:** Differences of mix design between batches. Fine aggregates are defined as the aggregates with size until 4 mm, the coarse ones are the rest of the aggregates.

Batch ID	D1	D2	D3
Water content	166.4 kg/m${}^{3}$	169.0 kg/m${}^{3}$	168.9 kg/m${}^{3}$
Cement content	275.8 kg/m${}^{3}$	275.0 kg/m${}^{3}$	280.0 kg/m${}^{3}$
Fine Aggregates	870.8 kg/m${}^{3}$	1251.9 kg/m${}^{3}$	755.8 kg/m${}^{3}$
Coarse Aggregates	1031.6 kg/m${}^{3}$	742.7 kg/m${}^{3}$	1322.2 kg/m${}^{3}$
Total Aggregates	1902.4 kg/m${}^{3}$	1994.6 kg/m${}^{3}$	2078.0 kg/m${}^{3}$
Water—Cement ratio	0.603	0.615	0.603
Aggregate—Cement ratio	6.90	7.25	7.42
Aggregate type	Quartz	Limestone	Basalt
Aggregate shape	Round	Round	Broken
Max. Aggregate Diameter	16 mm	16 mm	22 mm

**Table 2:** Compressive strength, tensile strength, elastic modulus, fracture energy and pull-out tests results for the different batches

Age	Property	Curing	D1	D2	D3
28 days	$f_{c}$ (MPa)	D	41.9±3.0%	32.4±6.4%	35.7±2.7%
	$f_{c}$ (MPa)	M	51.0±0.8%	40.2±6.2%	39.6±1.0%
	$f_{t,sp}$ (MPa)	M	3.62±2.9%	3.45±3.9%	3.00±6.0%
	*E* (GPa)	M	33.4±7.1%	29.1±10.4%	33.9±8.7%
	$G_{F}$ (N/m)	M	−	−	−
	$G_{f}$ (N/m)	M	−	−	−
	$N_{no}$ (kN)	D	86.7±5.6%	78.8±3.46%	82.4±5.7%
70 days	$f_{c}$ (MPa)	D	47.0±2.8%	40.3±6.2%	40.5±2.7%
	$f_{c}$ (MPa)	M	55.1±4.2%	38.9±5.3%	45.5±4.3%
	$f_{t,sp}$ (MPa)	M	4.19±6.6%	3.47±12.9%	3.35±12.2%
	*E* (GPa)	M	35.4±4.7%	35.6±8.0%	35.4±6.5%
	$G_{F}$ (N/m)	M	119.8±7.2%	86.8±14.2%	129.9±25.8%
	$G_{f}$ (N/m)	M	77.7±7.2%	55.8±13.9%	67.8±22.1%
	$N_{no}$ (kN)	D	103.5±5.9%	86.2±2.7%	95.0±2.8%
